# The association between body mass index and fibromyalgia severity: data from a cross-sectional survey of 2339 patients

**DOI:** 10.1093/rap/rkab015

**Published:** 2021-03-01

**Authors:** Fabiola Atzeni, Alessandra Alciati, Fausto Salaffi, Marco Di Carlo, Laura Bazzichi, Marcello Govoni, Giovanni Biasi, Manuela Di Franco, Flavio Mozzani, Elisa Gremese, Lorenzo Dagna, Alberto Batticciotto, Fabio Fischetti, Roberto Giacomelli, Serena Guiducci, Giuliana Guggino, Mario Bentivegna, Roberto Gerli, Carlo Salvarani, Gianluigi Bajocchi, Marco Ghini, Florenzo Iannone, Valeria Giorgi, Sonia Farah, Sara Bonazza, Stefano Barbagli, Chiara Gioia, Noemi Giuliana Marino, Annunziata Capacci, Giulio Cavalli, Antonella Cappelli, Francesco Carubbi, Francesca Nacci, Ilenia Riccucci, Maurizio Cutolo, Luigi Sinigaglia, Piercarlo Sarzi-Puttini

**Affiliations:** 1 Rheumatology Unit, Department of Internal Medicine, University of Messina, Messina; 2 Department of Clinical Neurosciences, Hermanas Hospitalarias, Villa San Benedetto Menni Hospital, Albese con Cassano, Humanitas Clinical and Research Center, Como, Milan, Rozzano; 3 Rheumatology Clinic, Department of Clinical and Molecular Sciences, Università Politecnica delle Marche, Jesi, Ancona; 4 Rheumatology Unit, AOU Pisana, Pisa; 5 Rheumatology, Department of Medical Sciences, University of Ferrara and Azienda Ospedaliera-Universitaria S. Anna di Ferrara; 6 Rheumatology Unit, Department of Medical Sciences, Surgery and Neurosciences, University of Siena, Siena; 7 Rheumatology Unit, Department of Internal Medicine, Anesthesiological and Cardiovascular Sciences, Policlinico Umberto I, Sapienza University of Rome; 8 Internal Medicine and Rheumatology Unit, Azienda Ospedaliero-Universitaria di Parma, Parma; 9 Rheumatology Unit, Fondazione Policlinico Universitario A. Gemelli IRCCS, Rome; 10 Unit of Immunology, Rheumatology, Allergy and Rare Diseases (UnIRAR), IRCCS San Raffaele Scientific Institute, Vita-Salute San Raffaele University, Milan; 11 Rheumatology Unit, Internal Medicine Department, ASST Settelaghi, Varese; 12 Unit of Rheumatology, Department of Medical, Surgery and Health Sciences, ASUGI and Clinical University, University of Trieste; 13 Clinical Unit of Rheumatology, School of Medicine, University of L'Aquila; 14 Division of Rheumatology AOUC, Department of Experimental and Clinical Medicine, University of Florence, Florence; 15 Department of Health Promotion Sciences, Maternal and Infant Care, Internal Medicine and Medical Specialties, University of Palermo, Palermo; 16 Integrated Reference Center of Rheumatology, Scicli Hospital, Ragusa; 17 Rheumatology Unit, Department of Medicine, University of Perugia, Perugia; 18 University of Modena and Reggio Emilia, Azienda USL-IRCCS di Reggio Emilia; 19 Rheumatology Unit, S. Maria Hospital—USL, IRCCS Institute, Reggio Emilia; 20 Rheumatology Unit, Azienda USL di Modena; 21 Rheumatology Unit, Department of Emergency Surgery and Organ Transplantations, University of Bari, Bari; 22 Rheumatology Unit, Internal Medicine Department, ASST Fatebenefratelli-Sacco, Milan University School of Medicine, Milan; 23 Research Laboratory and Division of Clinical Rheumatology, Department of Internal Medicine, University of Genoa, IRCCS San Martino, Genova; 24 Division of Rheumatology, ASST Gaetano Pini-CTO, Milano, Italy

**Keywords:** BMI, fibromyalgia, obesity, widespread pain, clinical severity

## Abstract

**Objective:**

Various studies have shown that overweight and obesity are central features of FM, but the real impact of a high BMI on clinical severity in patients with FM is still controversial. The aim of this study was to analyse the relationships between BMI categories and measures of symptom severity and functional impairment using data from a Web-based registry of patients with FM.

**Methods:**

Adult patients with an ACR 2010/2011 diagnosis of FM underwent a complete physical examination and laboratory tests and were asked to complete a package of questionnaires covering their sociodemographic and treatment details, in addition to the following disease-specific questionnaires: the revised Fibromyalgia Impact Questionnaire (FIQR), the modified Fibromyalgia Assessment Status questionnaire (ModFAS) and the Polysymptomatic Distress Scale (PDS).

**Results:**

A total of 2339 patients were recruited and divided into two weight categories, underweight/normal (U/N, *n* = 1127, 48.2%) and overweight/obese (O/O, *n* = 1212, 51.8%). The total and subscales of FIQR, ModFAS and PSD scores were significantly higher in the O/O patients, as were all the mean scores of the individual FIQR items (*P* < 0.001 for all).

**Conclusion:**

Our findings demonstrate that O/O patients with FM are significantly more impaired than U/N patients in all the symptomatological and functional domains as measured using the FIQR, ModFAS and PDS, thus suggesting that being O/O has an additional effect on symptoms and function.

**Key messages**

- Overweight/obesity is a co-morbidity present in more than half of FM patients.
- The presence of overweight/obesity in FM patients is associated with increased symptom severity and impaired function.

## Introduction

FM is characterized by widespread pain, fatigue, sleep disturbances and impaired cognitive function and is associated with depression and bipolar spectrum disorders, but its aetiology is unknown [1].

Over the last 20 years, increasing evidence has emerged that indicates a relationship between a high BMI, pain [2, 3] and painful syndromes such as FM [4]. One community-based twin registry study has shown that low back pain, abdominal pain, chronic widespread pain, headache and FM were more likely to be reported by overweight (BMI >25 but <30 kg/m^2^) and obese twins (BMI >30 kg/m^2^) than in their normal-weight counterparts [5]. Furthermore, the co-existence of a high BMI and FM has been demonstrated by an Internet-based survey [6] showing that 27% of >2500 patients with FM were overweight and 43% were obese, and a 2002 study of women with FM referred to a rheumatology clinic found that the prevalence of overweight/obesity was 61%, which is much higher than the 38% in the general population at the time [4]. More recent studies have reported similar or higher prevalence rates of 21–30% for overweight, and 43.8–50% for obesity [7–9].

The mechanisms underlying the relationship between a high BMI and FM are still unclear, but it has been suggested that the reduction in physical activity induced by musculoskeletal pain might lead to a higher BMI, or that a higher BMI causes pain as a result of increased strain on weight-bearing joints. Other possible explanations are that obesity and FM are both associated with the same alterations in endocrine function, opioid systems and inflammatory pathways [10], and this might affect the sensitivity to pain of obese patients with FM [11]. A recent study in patients with FM showed an association between BMI and increased cross-sectional area of the sural nerve as a possible expression of small-fibre neuropathy [12].

Psychological and psychiatric factors might also contribute to the relationship between obesity and FM. Various studies have shown that FM and obesity [13, 14] are both frequently associated with lifetime major depression [15] and, in particular, with bipolar spectrum disorders [1]. It is worth noting that both treated and untreated patients with bipolar disorders [16] weigh significantly more than controls [17] and are as much as four times more frequently obese or overweight [18].

A relationship has also been found between obesity and the clinical and biological characteristics of FM, although the results of studies aimed at evaluating the impact of increased BMI or overweight/obesity on FM symptom severity, disease activity and functional impairment are inconsistent [19–25].

The inconsistencies in the findings described above might be attributable to differences in the sociodemographic characteristics of the study samples and/or the assessment of FM symptoms, in addition to whether the patients were or were not divided into categories on the basis of the degree of obesity, which indicates that further studies are required to analyse the associations between obesity and FM. The aim of this study was to examine the relationship between BMI categories and measurements of FM-related symptoms and functioning in a large, multicentre cohort of patients with FM.

## Methods

### Subjects

The study involved adult patients aged 18–80 years with FM diagnosed on the basis of the 2010/2011 criteria of the ACR [26], who were recruited between November 2018 and April 2019 at 19 Italian rheumatology centres. At each centre, the diagnosis was made by a rheumatologist with ≥10 years of experience. All the patients underwent a complete physical examination and the laboratory tests specified in the revised EULAR recommendations for the management of FM [27]. The exclusion criteria were as follows: cardiovascular disease; moderate/severe chronic lung disease; uncontrolled hypertension; uncontrolled thyroid disorders; orthopaedic or musculoskeletal conditions prohibiting moderately intense exercise; inflammatory rheumatic conditions or other connective tissue diseases; and significant psychiatric conditions that would interfere with the assessment of FM, including severe depression and psychosis.

All the participants gave their written informed consent to the study. The protocol and the patient information sheet and consent form were approved by the Ethics Committee of the Università Politecnica delle Marche, Ancona, Italy (Comitato Unico Regionale—ASUR Marche, no. 1970/AV2), and the review boards of all the study centres. The study protocol did not require any medical intervention.

### Measurements

The data and measures described below were entered electronically into the Web-based Italian Fibromyalgia Registry by the physicians working at the 19 Italian rheumatology centres.

The patients were asked to complete a package of questionnaires covering their sociodemographic data (age, sex, marital status, education and BMI), disease-related variables, their quality of life, and the type(s) of pharmacological and non-pharmacological treatments currently received. Three disease-specific questionnaires were used for the clinical evaluation: the revised Fibromyalgia Impact Questionnaire (FIQR) [28], the modified Fibromyalgia Assessment Status questionnaire (ModFAS) [29] and the Polysymptomatic Distress Scale (PDS).

#### Revised Fibromyalgia Impact Questionnaire (FIQR)

The FIQR is the updated version of the Fibromyalgia Impact Questionnaire (FIQ) [30]. It consists of 21 items, 11-point numerical rating scales (NRS) (0–10) that investigate three main domains in relationship to the previous week: FM symptoms (10 items), physical function (9 items) and overall impact (2 items). The final score can range from 0 to 100 (higher scores indicate more severe disease) and is calculated as the algebraic sum of the symptoms domain divided by two, plus the physical function domain divided by three, plus the two items of the overall impact domain [28].

#### Modified Fibromyalgia Assessment Status (ModFAS)

The ModFAS is a revised and easier to use version of the Fibromyalgia Assessment Status questionnaire (FAS) [29] divided into two sections that investigate symptoms over the previous 7 days. The first section consists of two numerical rating scales ranging from 0 (no problem) to 10 (severe problem) that investigate fatigue and unrefreshing sleep; the second section asks patients to indicate which of 19 body areas was painful on the front and back of a drawn manikin. The final score can range from 0 to 39 and is the sum of the two NRS plus the manikin score.

#### Polysymptomatic Distress Scale (PDS)

The PDS is derived from the variables used in the 2010/2011 ACR diagnostic criteria for FM [26]. The PDS score is obtained by summing the scores of the widespread pain index (WPI; a 0–19 count of painful non-articular body regions) and the symptom severity scale (a 0–12 measure of the severity of the three symptoms of fatigue, sleep and cognitive problems) and ranges from 0 to 31, with higher scores indicating more severe disease.

### Statistical analysis

All the data were entered into a Microsoft Excel data management database and were analysed using 64-bit MedCalc, v.19.0.1.0 (MedCalc Software, Mariakerke, Belgium). The patients were stratified into BMI categories, expressed in kilograms per square metre, using the international criteria of underweight (<18.5), normal weight (18.5–24.9), overweight (25.0–29.9) and obese (>30.0). Given that there were only 68 patients who were underweight (2.9% of the study population), they were combined with the subjects with normal weight to form an underweight/normal weight (U/N) group; furthermore, the overweight and obese subjects were combined to form an overweight/obese (O/O) group for analytical purposes. Normal data distribution was verified using the Shapiro–Wilk test, and the data are presented as median values and interquartile range or mean values and s.d., as appropriate. Differences in sociodemographic characteristics between the two groups were analysed by means of the χ^2^ test and the one-way analysis of variance. Spidergrams were used to provide a graphical representation of the differences between the groups by weight status [31], and the association between weight status and study outcomes was examined by means of one-way analysis of covariance after adjusting for age. When the differences were significant, pairwise comparisons with Bonferroni’s adjustment were used to keep the experimental error rate at ≤0.05 and identify between which groups the differences were significant.

## Results

The data relating to 2339 patients [2181 women and 158 men, with a mean (s.d.) age of 51.91 (11.5) years at the time of enrolment] were entered into the Italian Fibromyalgia Registry between November 2018 and December 2019. In Table 1, the sociodemographic characteristics between the U/N and O/O groups are described. Beyond BMI, the main difference between the two groups was age, with the O/O group representing a slightly older population.

**Table 1:** Sociodemographic characteristics between the two groups of patients with FM divided according to the BMI

Characteristic	Total	Underweight/normal weight	Overweight/obese	*P*-value
Number of patients, *n* (%)	2339	1127 (48.2)	1212 (51.8)	–
Females/males, *n* (%)	2181 (93.24)/158 (6.76)	1052 (48.24)/75 (47.47)	1129 (51.76)/83 (52.53)	0.035*
Age, mean (s.d.), years	51.91 (11.52)	50.73 (11.66)	53.14 (11.26)	0.001^†^
Education, *n* (%)				0.009*
Primary school	155 (6.60)	58 (37.41)	97 (62.59)	
Middle school	660 (28.20)	309 (46.81)	351 (53.19)	
High school/university	1524 (65.29)	760 (49.86)	764 (50.14)	
Marital status, *n* (%)				0.003*
Single	413 (17.70)	222 (53.75)	191 (46.25)	
Married	1667 (71.30)	775 (46.49)	892 (53.51)	
Separated/divorced	200 (8.60)	105 (52.50)	95 (47.50)	
Widowed	59 (2.50)	25 (42.37)	34 (57.62)	
Age at onset of FM, mean (s.d.), years	42.8 (12.43)	41.82 (9.46)	43.788 (8.93)	0.028^†^
Duration of FM, mean (s.d.), years	7.34 (5.00)	7.31 (7.26)	7.36 (6.60)	0.846^†^
BMI, mean (s.d.), kg/m^2^	25.90 (4.20)	22.42 (2.17)	29.13 (3.70)	<0.001^†^
Weight categories, *n* (%)				
Underweight (BMI <18.5 kg/m^2^)	68 (2.9%)	68 (2.9%)	–	
Normal weight (BMI 18.5–24.9 kg/m^2^)	1059 (45.3%)	1059 (45.3%)		
Overweight (BMI 25–29.9 kg/m^2^)	891 (38.1%)	–	891 (38.1%)	
Obese (>30.0 kg/m^2^)	321 (13.7%)		321 (13.7%)	

* *P*<0.05, χ^2^ test. ^†^*P*<0.05, one-way analysis of variance.

**Fig. 1:**
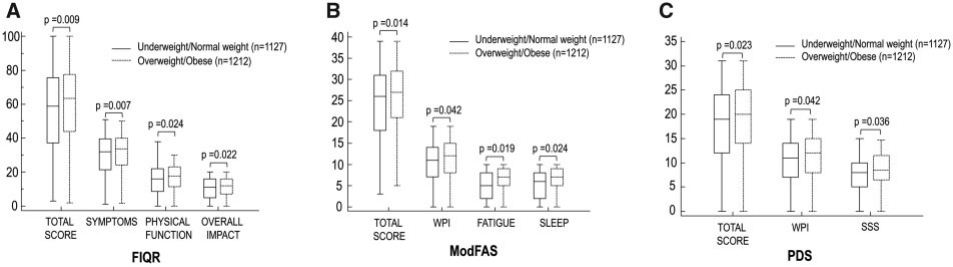
Differences in the scores of revised Fibromyalgia Impact Questionnaire (**A**), modified Fibromyalgia Assessment Status (**B**), Polysymptomatic Distress Scale (**C**) and respective subscales FIQR: revised Fibromyalgia Impact Questionnaire; ModFAS: modified Fibromyalgia Assessment Status; PDS: Polysymptomatic Distress Scale.

The total and three domain scores of the FIQR (physical function, overall impact and symptoms) were significantly higher in the O/O group (*P* = 0.009, *P* = 0.007, *P* = 0.024 and *P*  = 0.22, respectively; Fig. 1A), and the same was true of the total and three domain scores of the ModFAS (fatigue, quality of sleep and non-articular pain; *P*  = 0.014, *P*  = 0.019, *P*  = 0.024 and *P*  = 0.042, respectively; Fig. 1B) and the total PSD and WPI and symptom severity scale scores (*P*  = 0.023, *P* = 0.042, *P*  = 0.036, respectively; Fig. 1C).

Table 2 summarizes the values in terms of mean (s.d.) and median (interquartile range) of the three clinimetric indices (and subscales) distinguished in the two groups U/N *vs* O/O.

**Table 2:** Revised Fibromyalgia Impact Questionnaire, modified Fibromyalgia Assessment Status and Polysymptomatic Distress Scale scores in underweight/normal weight and overweight/obese patients with FM

Score	Underweight/normal weight (*n* = 1127)				Overweight/obese (*n* = 1212)			
	Mean	Median	s.d.	IQR	Mean	Median	s.d.	IQR
FIQR								
Total score (0–100)	55.90	58.83	24.04	37.20–75.66	59.69	63.33	22.59	43.91–77.50
Physical function (0–30)	15.32	16.00	7.79	8.66–22.00	16.76	17.66	7.61	11.33–23.00
Overall impact (0–20)	10.75	11.00	6.17	5.00–16.00	11.32	12.00	5.90	7.00–16.00
Symptoms (0–50)	29.82	32.00	11.83	21.50–39.50	31.58	33.75	11.01	24.50–40.00
ModFAS								
Total score (0–39)	21.47	22.00	8.44	13.00–25.00	24.31	26.00	9.76	15.00–29.00
Fatigue (0–10)	5.17	5.00	3.06	2.00–8.00	6.50	7.00	3.02	5.00–9.00
Sleep (0–10)	5.52	6.00	3.04	2.00–8.00	6.44	7.00	2.97	5.00–9.00
WPI (0–19)	10.78	11.00	4.92	7.00–14.00	11.37	12.00	4.86	8.00–15.00
PDS								
Total score (0–31)	18.08	19.00	7.50	12.00–24.00	19.07	20.00	7.213	14.00–25.00
SSS (0–12)	7.32	8.00	3.58	5.00–10.00	7.70	8.00	3.41	6.00–11.00
WPI (0–19)	10.78	11.00	4.92	7.00–14.00	11.37	12.00	4.86	8.00–15.00

FIQR: revised Fibromyalgia Impact Questionnaire; IQR: interquartile range; ModFAS: modified Fibromyalgia Assessment Status; PDS: Polysymptomatic Distress Scale; SSS: symptom severity scale; WPI: widespread pain index.

All the mean scores of the individual FIQR items were higher in the O/O group (*P* < 0.001 for all; Table 3). The highest scoring items in the O/O group (i.e. the symptoms that had the greatest impact) were those related to the core symptoms of FM: pain (FIQR-12: mean score 7.08), fatigue/energy (FIQR-13: mean score 7.48), sleep quality (FIQR-15: mean score 7.42) and tenderness (FIQR-19: mean score 6.96). Likewise, the highest scoring items in the U/N group also related to the core symptoms, but the scores were significantly lower than those in the O/O group. In both groups, the lowest scoring items included functional activities, such as brushing/combing hair (FIQR-1: mean scores 4.07 and 0.93) and preparing a home-made meal (FIQR-3: mean scores 4.65 and 1.36).

**Table 3:** Mean scores for each revised Fibromyalgia Impact Questionnaire item in the underweight/normal weight and overweight/obese groups

Item	Item description	Underweight/normal weight		Overweight/obese	
		Score	s.d.	Score	s.d.
FIQR-1	Brush or comb hair	0.93	1.82	4.07	3.18
FIQR-2	Walk continuously for 20 min	1.77	2.25	5.36	3.02
FIQR-3	Prepare a home-made meal	1.36	1.59	4.65	2.96
FIQR-4	Vacuum, scrub or sweep floors	3.23	2.49	6.67	2.46
FIQR-5	Lift and carry a bag full of groceries	3.01	2.53	6.95	2.52
FIQR-6	Climb one flight of stairs	2.13	1.92	5.09	2.76
FIQR-7	Change bed sheets	2.72	2.46	6.49	4.52
FIQR-8	Sit in a chair for 45 min	3.24	2.56	5.96	2.81
FIQR-9	Go shopping for groceries	1.82	1.88	5.69	2.72
FIQR-10	Cannot achieve goals	2.09	1.87	5.99	2.42
FIQR-11	Feel overwhelmed	2.24	2.20	6.12	2.54
FIQR-12	Pain rating	3.42	2.46	7.08	2.00
FIQR-13	Fatigue rating	3.80	2.63	7.48	2.28
FIQR-14	Stiffness rating	3.56	2.43	6.86	2.14
FIQR-15	Sleep quality	3.90	2.74	7.42	2.29
FIQR-16	Depression level	2.44	2.21	5.80	2.92
FIQR-17	Memory problems	2.51	2.18	5.49	2.61
FIQR-18	Anxiety level	3.06	2.36	5.86	2.92
FIQR-19	Tenderness level	3.72	2.61	6.96	2.25
FIQR-20	Balance problems	2.44	2.21	5.53	2.41
FIQR-21	Environmental sensitivity	3.77	2.96	6.36	2.40

FIQR: revised Fibromyalgia Impact Questionnaire.

**Fig. 2:**
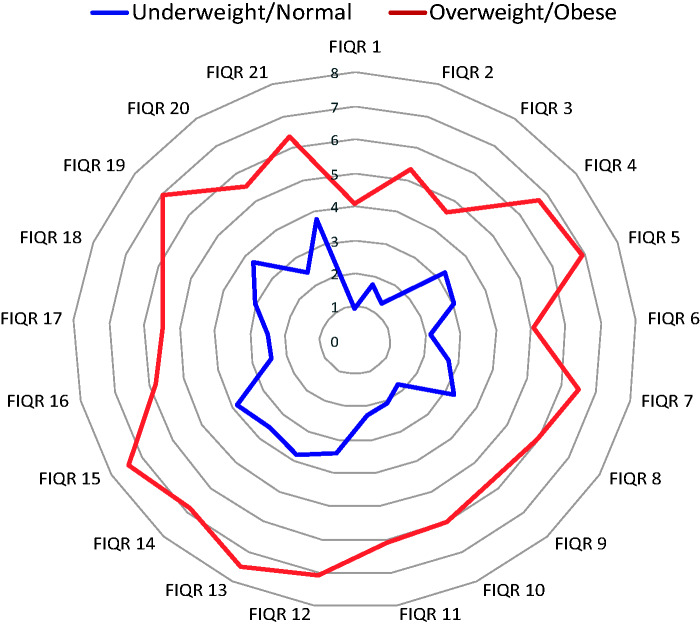
Spidergram with graphical representation of the differences in the 21 revised Fibromyalgia Impact Questionnaire subscales FIQR: revised Fibromyalgia Impact Questionnaire.

To compare the mean scores of the individual FIQR items between the O/O and U/N groups, a spidergram was generated, with the FIQR domain scores plotted from zero (best) at the centre to eight (worst) at the outer edge (*P* < 0.001 for all; Fig. 2).

## Discussion

Our findings show that being obese/overweight has an additional impact on the symptoms and function of patients with FM, leading to significantly greater impairment as measured using the FIQR, the ModFAS and the PDS. The findings are based on a real-world analysis of the largest population that has ever been studied clinically with the aim of evaluating the association between a high BMI and the clinical manifestations and functioning of patients with FM. The 58% prevalence of overweight/obesity in the study population and the mean BMI of 25.11 kg/m^2^ were similar to those observed in another European population [32], but lower than those observed in studies carried out in the USA [8, 33], which is in line with epidemiological evidence showing that the prevalence of severe or extreme obesity is increasing at a faster rate than moderate obesity among American adults [34].

Yunus *et al.* [4] found no correlation between BMI and pain or fatigue, sleeping difficulties, irritable bowel syndrome or self-reported measures of anxiety and depression in a sample of 211 females with FM, but did find a relationship with functional impairment as evaluated using the HAQ Disability Index. Likewise, another study of 224 patients with FM [7] found that obesity was not correlated with FM symptoms or measures, including the total and 10 subset scores of the FIQ, but was associated with more general measures of disability (HAQ Disability Index).

On the contrary, a study of 100 women with FM [9] observed significant negative correlations between BMI and the quality of life and tenderness threshold, and significant positive correlations with physical dysfunctioning and tender point counts, and a similar trend was observed when these measures were compared in the three BMI categories of normal weight, overweight and obesity. These findings are in line with those of a study of 124 women with FM [19], which showed significant positive correlations between BMI and pain, tender point counts, FIQ and Hamilton Depression Scale scores, and significant differences in these measures between normal weight, overweight and obese patients, with the highest scores being found among the obese; no significant difference in anxiety levels was detected.

In agreement with these conclusions are studies showing that obesity was significantly correlated with greater pain sensitivity, tender point palpation, reduced physical strength and lower-body flexibility, shorter sleep duration and greater restlessness during sleep [8]; overweight and obese patients with FM had higher levels of pain, fatigue, morning tiredness and stiffness in comparison with their normal weight counterparts [20]; severely obese patients had significantly greater FM-related symptoms and a poorer quality of life than non-obese or overweight patients [21]; a higher BMI was associated with poor FIQR scores [22]; and, finally, total and central body fat were positively associated with pain‐ and fatigue‐related measures and total FIQR scores [23].

A study of 177 obese women with FM [24] did not find any differences in FM symptoms or quality of life dimensions between different categories of obesity (obesity I: BMI 30.0–34.99 kg/m^2^; obesity II: BMI 35.0–39.99 kg/m^2^; and obesity III: BMI ≥40.0 kg/m^2^), thus suggesting that the obesity-related impairments observed in patients with FM are not related to the degree of obesity.

Finally, in another recent study of 34 postmenopausal women with FM and 22 healthy controls classified on the basis of their BMI [25], the patients with FM reported worse dynamic and static balance, poorer functional mobility and higher levels of physical disability regardless of their nutritional status, which suggests that BMI probably does not play a major role in the impaired functional capacity of postmenopausal patients with FM.

Our findings support the view that obesity/overweight is a significant co-morbidity in patients with FM. In addition to having a negative association with symptom severity and functional impairment, it might also affect therapeutic processes. Together with pharmacological treatment and cognitive behavioural therapy, aerobic exercise is one of the cornerstones of FM treatment [27], and obese patients are significantly less fit than their non-obese counterparts, as shown by their shorter treadmill walking distance and higher maximum heart rate [10]. Furthermore, protracted periods of exercise can have beneficial effects on fatigue and chronic widespread pain in patients with FM [35], but a study of a group of women with FM has shown that these responses are delayed in overweight and obese patients [36].

Likewise, a study of subjects with chronic low-back pain [37] has shown that the effects of cognitive behavioural therapy on measures of disability, emotional functioning and the physical aspects of the quality of life were less positive in the obese than the non-obese participants. A more recent randomized controlled study has suggested that obesity has a negative impact on the efficacy of motivational interviewing (an approach aimed at modifying a number of health risk behaviours) in improving global FM symptom severity and pain [38]. However, it has also been found that the response to a multidisciplinary FM treatment programme that combines pharmacological treatment, education, physical therapy and cognitive behavioural therapy was not affected by differences in the BMI [39].

Given the negative association between obesity and FM severity, it seems that weight loss programmes should be given a central role in the approach to obese patients with FM. One study found that patients who lost an average of 4.4% of their initial weight during a 20-week programme of behavioural weight loss treatment achieved improvements in FM symptoms, pain interference, body satisfaction and the quality of life [40]. Likewise, a randomized controlled trial reported that weight loss in obese patients with FM led to significant improvements in FIQ scores, depression, sleep quality and tender point counts [41]. However, an important issue is how to maintain the weight loss and its benefits. Relapse and regained weight are common after obesity surgery and behaviourally oriented weight management programmes [42] because both of these treatments require the establishment of adaptive eating and activity habits that are difficult to maintain even in the case of healthy people. One study designed to identify barriers to weight management interventions in obese women with FM [43] has found that FM women have complex views about their bodies and specific needs that must be considered when developing weight management programmes and post-treatment approaches to the maintenance of weight loss.

Our study has a number of limitations. Firstly, its cross-sectional design precludes postulating causal relationships between a high BMI and the severity of FM symptoms and functional impairment; therefore, future studies should preferably have longitudinal designs in order to allow causal inferences to be made. Secondly, our patients came from tertiary care clinics, and our findings might not apply to all patients with FM. Thirdly, the study considered only the association between obesity/overweight and FM, excluding the other co-morbidities frequently associated with a high BMI (e.g. cardiovascular disease, chronic lung disease or depression); given that it excluded subjects with cardiovascular or pulmonary disease, the moderating effects of these two conditions could not be evaluated, and depression was measured using the FIQR depression subscale and not by means of a validated depression-specific questionnaire. Finally, the study included only patients with FM. Future studies should also include subjects without FM in order to be able to evaluate any differences or similarities.

However, one strength of the study is that the data were obtained in naturalistic settings from consecutive patients with FM who underwent standardized, comprehensive investigations (clinimetric evaluations, a physical examination and laboratory tests) and included demographic and medication data.

In conclusion, our findings suggest that overweight/obesity plays a role in the clinical and functional outcomes of FM and support the view that future studies investigating the mechanisms underlying the effects of BMI on outcomes and potential strategies for achieving and maintaining weight loss would be useful.

*Funding*: No specific funding was received from any bodies in the public, commercial or not-for-profit sectors to carry out the work described in this article.

*Disclosure statement*: The authors have declared no conflicts of interest.

## Data availability statement

Data are available upon reasonable request to the corresponding author.
